# Failed spinal anesthesia due to accidental dural puncture: a case report

**DOI:** 10.1186/s40981-022-00579-4

**Published:** 2022-10-29

**Authors:** Jun Honda, Yuki Yamazaki, Tatsumi Yakushiji, Hinako Hirata, Satoki Inoue

**Affiliations:** 1 Department of Anesthesiology, Fukushima Medical University Hospital, 1 Hikarigaoka, Fukushima, Fukushima 960-1295 Japan; 2 Department of Anesthesiology, Iwaki City Medical Center, 16, Kusehara, Mimaya-machi, Uchigo, Iwaki, Fukushima, 973-8555 Japan

**Keywords:** Accidental dural puncture, Spinal anesthesia, Combined spinal-epidural anesthesia, Cerebrospinal fluid, Failed spinal anesthesia

## Abstract

**Background:**

We present a case of inadequate spinal anesthesia possibly due to cerebrospinal fluid (CSF) leakage into the epidural space caused by accidental dural puncture (ADP).

**Case presentation:**

A 28-year-old woman with twin pregnancy underwent a cesarean section. She was scheduled to undergo combined spinal-epidural anesthesia (CSEA). Hyperbaric bupivacaine 9 mg with fentanyl 15 μg, with an additional bupivacaine 5 mg was administered from the L3/4 interspace for spinal anesthesia after repeated ADP at T12/L1; however, analgesia level was only up to T12. Insufficient analgesia level would be attributed to leakage of bupivacaine into the epidural space with the CSF via the injured dura. Planned surgery was performed under general anesthesia and completed uneventfully.

**Conclusion:**

In spinal anesthesia performed after ADP in pregnant women, the anesthesia level may not increase as expected if there is a large amount of CSF leakage.

## Background

Accidental dural puncture (ADP) is a complication of epidural anesthesia and a cause of post-dural puncture headache (PDPH), which is thought to be caused by cerebrospinal fluid (CSF) leakage, although the pathogenesis is not clear. Here, we present a case in which a spinal anesthesia level was not increased, possibly due to CSF leakage to the epidural space after ADP. Written informed consent for this case report was obtained from the patient.

## Case presentation

A 28-year-old woman (165 cm in height, and 71 kg in weight [53 kg when not pregnant]) with twin pregnancy was scheduled for a cesarean section under CSEA at our hospital.

An attempt was made to place an epidural catheter from T12/L1 in the left lateral position using the loss of resistance technique with an 18 G Tuohy needle and saline. However, when the Tuohy needle was advanced about 5 cm with median approach, ADP occurred and CSF flowed vigorously. Next, an epidural catheter was attempted to be placed from T11/12, but CSF leakage was observed slowly from the Tuohy needle. Another puncture was made at T11/12, but CSF leakage was also observed, so the epidural catheter was not placed. Spinal anesthesia was performed from L3/4 using a 25-G pencil point needle. After confirming CSF reflux in the usual manner of regurgitation approximately at 5.5 cm of the needle depth, 1.8 ml of 0.5% hyperbaric bupivacaine and 15 μg (0.3 ml) of fentanyl were administered. The patient was returned to the supine head-down tilt position, but after 8 min the anesthesia level had only increased to T12. The patient was then repositioned into the left lateral position for another dose of spinal anesthesia, and an additional 1 ml of 0.5% hyperbaric bupivacaine was administered from L3/4. However, after 18 min, the patient still felt pain in the lower abdomen, so the operation was finally performed under general anesthesia. Postoperative abdominal X-ray showed no lumbar deformity. The patient continued to have severe headaches for more than 3 weeks after surgery.

The patient was scheduled for another cesarean section 2 years later. Spinal anesthesia only was administered with 2 ml of 0.5% hyperbaric bupivacaine and 15 μg (0.3 ml) of fentanyl using a 25-G pencil point needle from L3/4, this time achieving an anesthetic level of T5.

## Discussion

Analgesia level was supposed to spread widely after spinal anesthesia in this patient because of securely confirming backflows of CSF. However, analgesia was insufficient even after repeated administration of bupivacaine and there would be several reasons for this. Bupivacaine might have been administered outside of the subarachnoid space although a definite backflow of CSF was confirmed. The CSF collected in the epidural space after dural puncture might have been aspirated into the spinal needle inserted from L3/4 and bupivacaine was injected into the epidural space, based upon a misunderstanding that the tip of the spinal needle was properly advanced into the subarachnoid space. It would have been possible. However, such a condition could be denied in this case because the anesthesia level had increased to T12 after administration of drugs, which suggested that drugs were injected into the subarachnoid space and the backflow of CSF was certainly from the subarachnoid space. However, such a condition would hardly occur because even anesthetic solutions injected into the epidural space in clinical settings are not easily aspirated. Arachnoid cyst might have been one of the reasons for failed spinal anesthesia [1], which would not have been existed because there was no problem in spinal anesthesia performed 2 years later.

Another possibility is that bupivacaine administered into the subarachnoid space partly leaked into the epidural space, providing insufficient anesthesia level by remaining bupivacaine in the subarachnoid space. Hyperbaric bupivacaine spread cephalad according to gravity after the patient was placed on the supine position, and leaked out through the injured site of the dura. We suspect that relatively large amount of CSF leaked into the epidural space, judged from the severity of postoperative headache [2]. The CSF flowing from the Tuohy needle at T11/12 might have been that collected in the epidural space leaked through the injured site of dura because collection of the CSF in the epidural space after spinal tap is reported in children as well as in young adults [3, 4]. However, a lack of MRI or ultrasound image confirming the collection of the CSF in the epidural space is a limitation of this report.

Continuous spinal anesthesia has been reported to be useful for caesarean section [5]. Therefore, conversion of CSEA to continuous spinal anesthesia might have been an option for anesthesia management after the second ADP.

In conclusion, we experienced a case in which the level of anesthesia was not as high as expected for spinal anesthesia after ADP in a pregnant woman. Clinicians must keep in mind the possibility that spinal anesthesia may be inadequate after ADP in pregnant women due to leakage of CSF.

## Data Availability

Not applicable.

## Abbreviations

ADP: Accidental dural puncture
CSEA: Combined spinal-epidural anesthesia
PDPH: Post-dural puncture headache
CSF: Cerebrospinal fluid
